# Correlation of Sunlight Exposure and Different Morphological Types of Age-Related Cataract

**DOI:** 10.1155/2021/8748463

**Published:** 2021-11-12

**Authors:** Xiaochun Li, Xiaoguang Cao, Yingying Yu, Yongzhen Bao

**Affiliations:** ^1^Department of Ophthalmology, Peking University People's Hospital; Eye Diseases and Optometry Institute; Beijing Key Laboratory of Diagnosis and Therapy of Retinal and Choroid Diseases, College of Optometry, Peking University Health Science Center, 11# Xizhimen South Street, Xicheng District, Beijing 100044, China; ^2^Department of Ophthalmology, Peking University International Hospital, 1# Shengmingyuan Road, Life Science Park, Changping District, Beijing 102206, China

## Abstract

**Purpose:**

The previous lab and clinical studies of the correlation between the ultraviolet B and age-related cataract (ARC) did not reach in the universal agreement, especially in different morphological types of ARC. It is important to systemically summarize those previous data of epidemiological studies, which might penetrate the relevance between three morphological types of ARC, cortical, nuclear, and posterior capsular (PSC), with sunlight exposure.

**Methods:**

PubMed, Web of Science, CNKI, Embase, and Cochrane were searched online. Data were extracted and recalculated, and quality check was performed by hand. Review Manager was used to perform the fixed effects meta-analysis on ARC and its morphological types. The highest exposed dose group was defined as the exposed group, and the lowest dose group as the control group as possible.

**Results:**

Finally, the number of analyzed studies was 31: 20 for ARC and twelve, eleven, and nine for the morphological types cortical, nuclear, and PSC, respectively. The pooled OR for ARC was 1.15 (range 1.00~43.78, 95% CI 1.09 to 1.21). The cortical cataract revealed a slightly higher risk, and pooled OR was 1.03 (range 0.67~2.91, 95% CI 1.02 to 1.03). But the pooled OR for nuclear and PSC were 1.00 (range 0.50~5.35, 95% CI 1.00 to 1.00) and 0.99 (range 0.57~1.87, 95% CI 0.95 to 1.01), respectively.

**Conclusions:**

The systemic analysis of epidemiological articles reported till now reveals a significantly increased risk of ARC for those exposed with more sunlight, especially the morphological type of cortical cataract.

## 1. Introduction

As the leading cause of blindness many years worldwide, age-related cataract (ARC) still induces the bilaterally blindness on estimated 17 million individuals [1]. The report of China's Ministry of Health showed that there were approximately four million cataract patients in China, with a half million diagnosed new cases annually. As the only effective treatment, the cataract extraction might not get a good result for every patient, due to the inexperienced surgeons and inappropriate postoperative care and refractive error, and even could not be available at some time [2, 3]. Now, the cataract extraction could be the most frequent operation among Medicare beneficiaries similar in China (1042850 in 2009) and in the United States (3280966 operations in 2003 and 2004) [4]. Moreover, the China Disabled Persons' Federation reported that the cost of cataract surgeries was over three billion RMB each year. A previous study showed that a ten-year delay on the onset of cataract could reduce the half need of cataract extraction as much [5].

The etiology of cataract, the lens opacity, involved many factors. In these, ultraviolet radiation (UV) might be the most important one besides the aging. In animal models, UVB (UV at 280-315 nm) could induce lens opacity. But as the previous epidemiological studies in human, the universal agreement has not been drawn and the controversial results were reported, especially in the three different morphological types (cortical, nuclear, and posterior capsular) of ARC. The risk of cortical cataract increased with the increasing or accumulated amount of UVB radiation exposure, reported by some studies. However, the epidemiological studies for the nuclear type of ARC reported the mutually contradictory results. Unfortunately, the epidemiological studies of posterior capsular cataract (PSC) were quite less than the others; a few studies thought that the UVB might have an effect on PSC [6–14]. As different morphological types (cortical, nuclear, and posterior capsular) of ARC have an inequable effect on the patients' visual function, the exploration of cataract morphological types might contribute to the increase in patients' quality of life.

To end the debate, a systematic review and meta-analysis were performed to assess the association between sunlight exposure with ARC and its three morphological types. We assumed that the exposure of sunlight was a risk factor for ARC. The discrepancy between those previous studies grew out of the differences of study population and methodology.

## 2. Methods

### 2.1. Research Project

We conduct a meta-analysis on the previous epidemiological literatures of cataract, based on the meta-analysis of observational studies in epidemiology (MOOSE) [15]. CNKI, Web of Science, PubMed, Embase, and Cochrane Library databases (up to 9 September 2021) were searched online and by hand for published articles. IRB/Ethics Committee ruled that approval was not required for this study. The methodology was partly referred [16].

### 2.2. Principle of Included and Excluded Articles

Articles that met the criteria would be selected: epidemiological study focused or partly focused on the correlation of ARC and sunlight exposure, whose odds ratio (OR) and 95% CI could be extracted or recalculated. “UV exposure”, “visible light exposure”, and “blue light exposure” were considered the same as sunlight exposure. All studies, which only focused on the laboratory, such as case report, letter, and experimental study, would be excluded.

In the subtype cataract setting, the included studies were divided into ARC and its three morphological types, cortical cataract, nuclear cataract, and PSC. If the data in the included manuscript were represented as cortical cataract, nuclear cataract, or PSC, it would be used for the calculation of each subtype, cortical cataract, nuclear cataract, and PSC. If the data were mentioned as mixed cataract or “cataract” only, it would be used for the calculation of the ARC subtype.

### 2.3. Search Principle

There was no restriction about language. We searched PubMed, Web of Science, and CNKI using the terms “ARC”, “risk OR incidence OR epidemiologic”, and “sunlight OR UV OR ultraviolet OR blue light OR visible light”. The methodology was partly referred [16]. For the Embase and Cochrane Library databases, as the advanced search does not support the complicated terms, “Cataract AND visible light”, “Cataract AND blue light”, “Cataract AND sunlight”, and “Cataract AND ultraviolet” were searched separately and the results were evaluated manually paper by paper. All articles, which could be extracted OR or had sufficient data to calculate OR, would be included in our study.

### 2.4. Data Extraction and Calculation

OR of the sunlight exposed/unexposed and their 95% CI were extracted by one author (Xiaoguang Cao) from the included articles besides general information. We calculated the OR ourselves by the software, Review Manager 5.2.4 (Java 6) for the articles without presenting OR with the published data. The definition of dose groups was partly referred [16].

### 2.5. Quality Check

Two checklists were used for the methodological quality evaluation and separately for cross-sectional studies and cohort/case-control studies as those previous designs [16], respectively. The two checklists contain all 31 items pertaining to population selection, comparability, and ascertainment of outcomes as presented in Tables 1 and 2.

### 2.6. Statistical Analysis

The *Q*-test was used to explore the heterogeneity between included studies. The *Z*-test was used for the pooled OR statistical significance. The correlation of sunlight exposure and ARC was evaluated with OR and 95% CI. A *P* value of less than 0.05 was considered to be statistically significant. Meta-analysis and metaregression were both performed using Review Manager 5.2.4 (Java 6). The methodology was partly referred [16].

## 3. Results

In included studies, thirty-one studies were included during the year of 1980 to 2020.  Table 3 shows the included studies in ARC and three subtypes. Tables 1 and 2 show the quality evaluation of included articles.

**Figure 1:**
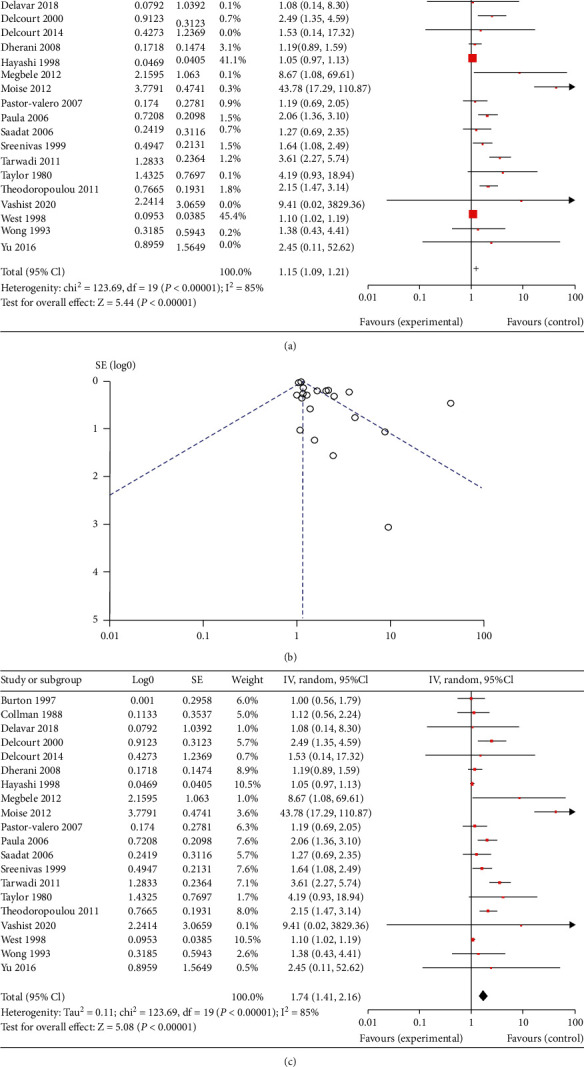
The result of meta-analysis for age-related cataract (ARC): forest plot of fixed effects meta-analysis (a); funnel plot of fixed effects meta-analysis (b); forest plot of random effects meta-analysis (c).

For the ARC, 20 studies were included [9, 17–35]. The results of fixed effects meta-analysis indicated a significantly increased risk of ARC in the more exposed group, with a pooled OR of 1.15 (range 1.00~43.78, 95% CI 1.09 to 1.21, as Figure 1(a). A forest plot with details is presented in Figure 1(a) is asymmetric. And the result of random effects meta-analysis indicated a pooled OR of 1.74 (95% CI 1.41 to 2.16) as shown in Figure 1(c).

### 3.1. Analysis of Subtype

**Figure 2:**
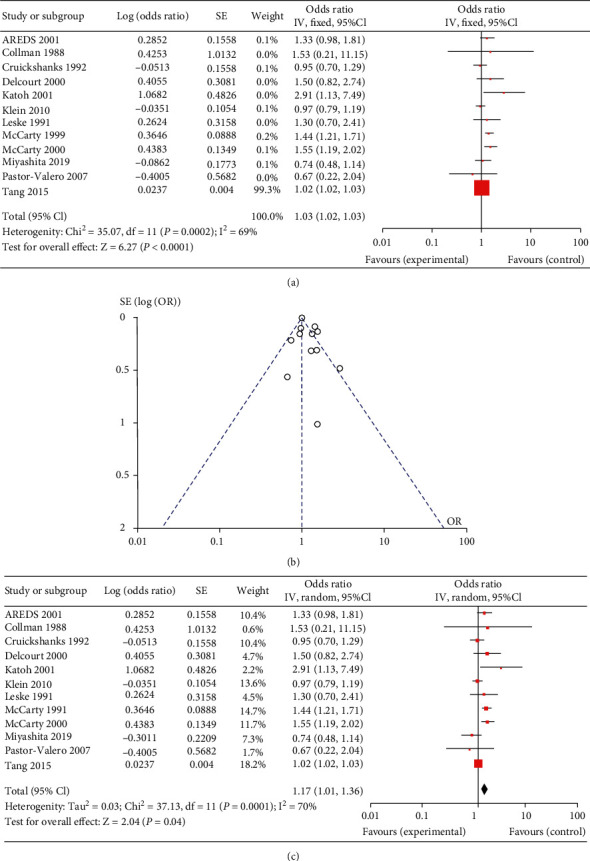
The result of meta-analysis for the subtype, cortical cataract of age-related cataract (ARC): forest plot of fixed effects meta-analysis (a); funnel plot of fixed effects meta-analysis (b); forest plot of random effects meta-analysis (c).

In cortical cataract, twelve studies were included [6, 8, 18, 19, 24, 36–42]. The results of the fixed effects meta-analysis indicated a slight increased risk of cortical cataract in the more exposed group than in other ARC subtype, with a pooled OR of 1.03 (range 0.67~2.91, 95% CI 1.02 to 1.03, as Figure 2(a). A forest plot with details is presented in Figure 2(a). The funnel plot (Figure 2(b)) is borderline asymmetric. And the result of random effects meta-analysis indicated a pooled OR of 1.17 (95% CI 1.01 to 1.36) as shown in Figure 2(c).

**Figure 3:**
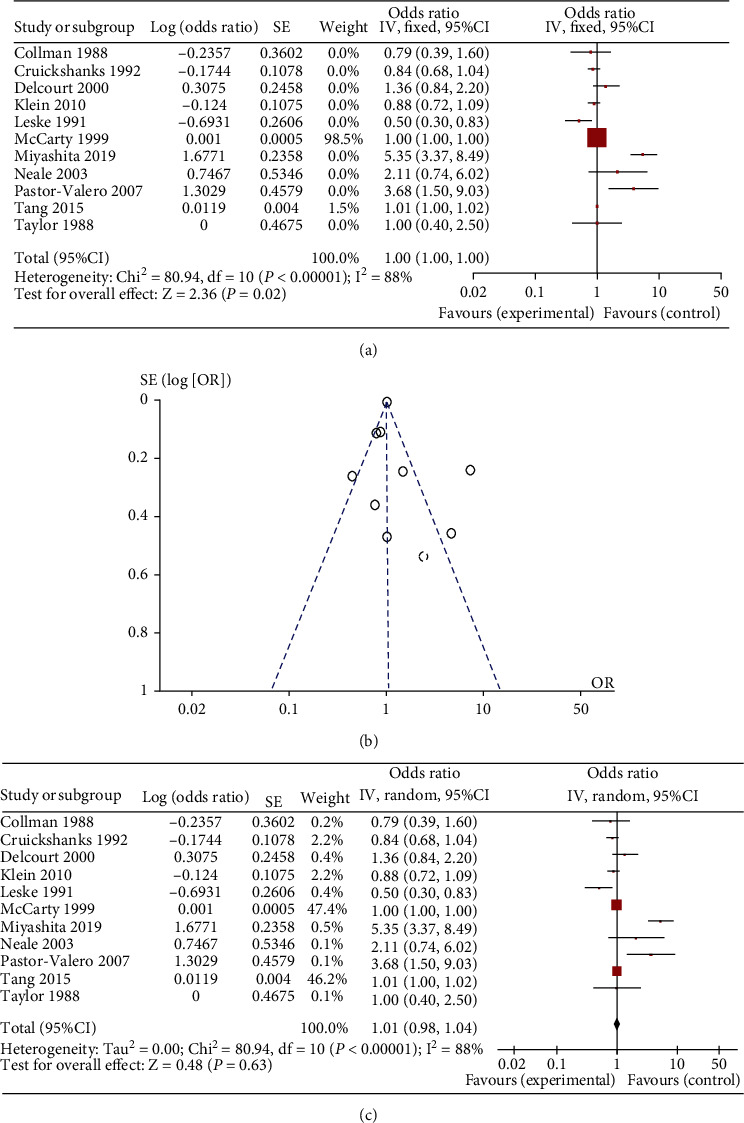
The result of meta-analysis for the subtype, nuclear cataract of age-related cataract (ARC): forest plot of fixed effects meta-analysis (a); funnel plot of fixed effects meta-analysis (b); forest plot of random effects meta-analysis (c).

In nuclear cataract, eleven studies were included [8, 10, 18, 19, 24, 37, 39–43]. The results of the fixed effects meta-analysis did not indicate a significantly increased risk of nuclear cataract in the more exposed group, with a pooled OR of 1.00 (range 0.50~5.35, 95% CI 1.00 to 1.00, Figure 3(a)). A forest plot with details is presented in Figure 3(a). The funnel plot (Figure 3(b)) is symmetric. And the result of random effects meta-analysis indicated a pooled OR of 1.01 (95% CI 0.98 to 1.04 as shown in Figure 3(c).

**Figure 4:**
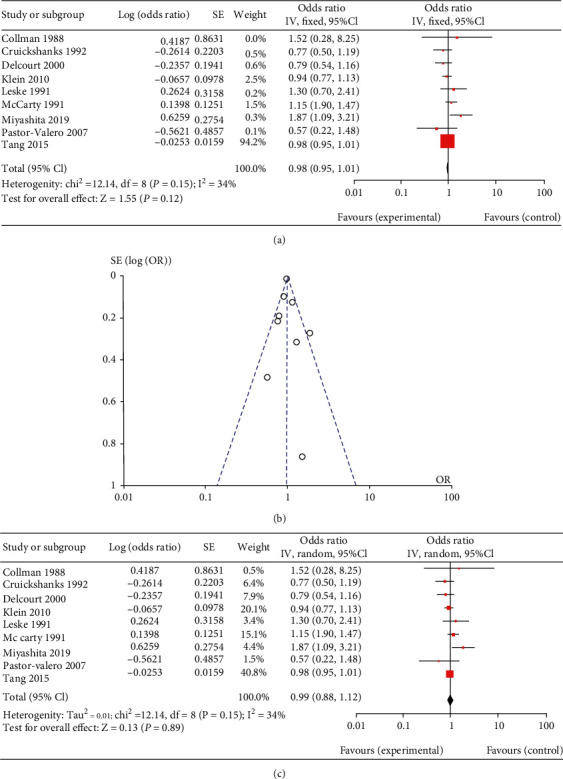
The result of meta-analysis for the subtype, posterior capsular cataract of age-related cataract (ARC): forest plot of fixed effects meta-analysis (a); funnel plot of fixed effects meta-analysis (b); forest plot of random effects meta-analysis (c).

In PSC, nine studies were included [8, 18, 19, 24, 37, 39–42]. The results of the fixed effects meta-analysis did not indicate a significantly increased risk of PSC in the more exposed group, with a pooled OR of 0.99 (range 0.57~1.87, 95% CI 0.95 to 1.01, as Figure 4(a)). A forest plot with details is presented in Figure 4(a). The funnel plot (Figure 4(b)) is symmetric. And the result of random effects meta-analysis indicated a pooled OR of 0.99 (95% CI 0.88 to 1.12) as shown in Figure 4(c).

## 4. Discussion

As the leading cause of blindness, the etiology of ARC keeps dimness. Although the experimental evidence of sunlight, especially UVB-induced damage of lens epithelial cell and crystallin, seemed sufficient as follows, epidemiological evidence was unstable. The involvement of oxidative stress in the development of cataract has been researched and discussed for years [44]. Many previous studies had shown that the ultraviolet radiation could induce the apoptosis of lens epithelial cells, through the cell sign pathway of caspase-3/Bcl-2/Bax [45]. Specific crystallins might play some important roles in the process [46]. Advance glycation end products and plasma membrane calcium ATPase1 are important contributors during cataract development [47].

Even numbers of epidemiologic studies on ARC have been published, and there is no meta-analysis to explore the association between ARC and sunlight exposure until our study, especially for different morphological types. The significant pooled OR of 1.15 (95% CI 1.09 to 1.21) is an evidence for our conjecture, in which the sunlight exposure would increase the prevalence of ARC.

ARC could be classified as three types in morphology, cortical, nuclear, and PSC [48], and cortical cataract counted as 60%-70% in ARC. Previous studies showed that the prevalence of ARC had obvious geographical difference. For example, the prevalence of ARC in people aged 40 years and older was 30% in Victoria, Australia, and the proportion of cortical cataract increased with the decreased latitude [6]. The study in Shunyi Beijing, China, showed that the prevalence of ARC in people aged 50 years and older was 23.31% [49].

Our study indicated that the sunlight exposure had a slightly higher risk on the cortical cataract in the three types of ARC (pooled OR 1.03, 95% CI 1.02 to 1.03). However, the sunlight exposure did not have influence on the nuclear cataract (pooled OR 1.00, 95% CI 1.00 to 1.00) or PSC (pooled OR 0.99, 95% CI 0.95 to 1.01).

The influence of sunlight exposure on cortical cataract is consistent with the previous epidemiological studies. Some studies already showed the risk of cortical cataract increased by the increasing amount of UVB radiation exposure, with a dose-dependent relationship [6–11]. At the same time, the damage of UVB on lens was a cumulative effect. McCarty and Taylor thought the development of cortical cataract associated with UVB [7]; the ocular UVB exposure could explain the 10% of cortical cataract [6]. Moreover, West et al. thought that not only was the risk of cortical cataract increased with UVB radiation but also the damage on lens was a cumulative effect [9]. The study of Taylor in Chesapeake Bay showed that in 838 watermen, the cataract patients had more 21% UVB exposure than the controls. UV radiation increases 1 time, and the risk of cortical cataract increased 1.6 times. The risk of cortical cataract for the highest radiation exposure in the investigated people was 3.3 times that for the lowest radiation exposure [10, 11].

For nuclear cataract, the results of epidemiological studies were mutually contradictory. McCarty et al. believed that the risk factors of nuclear cataract included ocular UVB exposure [8], whereas Taylor thought that UVB exposure had no effect on the nuclear cataract [10, 11]. The study performed by Hayashi et al. in Japan showed that the opacity of lens was related to the exposure dose of UVB, and the effect of UV on the nuclear cataract was stronger than that on cortical cataract [12]. One interesting study found a significant relationship between UV dosage and color naming: in low-UV localities, languages generally have the word “blue”; in high-UV areas, languages without “blue” prevail. It might be explained by the density change of lens induced by UV exposure [13]. Our study might end the debate, as a pooled OR of 1.00 (95% CI 1.00 to 1.00) indicated that the exposure of sunlight has no effect on the nuclear cataract, either harmful or protective.

The epidemiological studies of PSC were quite less than the others. McCarty and Taylor and Bochow et al. both thought that UVB radiation might have an effect on the incident of PSC [7, 14]. Our study showed that the pooled OR of sunlight exposure on PSC was 0.99 (95% CI 0.95 to 1.01), which indicated that the sunlight exposure had no effect on PSC.

Although the result of our study was consistent with the previous epidemiological studies of UVB exposure and incidence of ARC, it is premature to exclude UVA or even visible light in the etiology of ARC, especially cortical cataracts [50]. Exposure to intense artificial light and sunlight either caused or exacerbated age-related ocular diseases [51]. One previous study showed that long UVB and UVA might be involved in age-related alterations of the human lens and cataract formation [52]. The study by Zigman found that UVA might be easier inducing ARC than UVB [53].

As there is no universal accepted criterion for the definition of sunlight exposure, its effect on the prevalence of ARC cannot be calculated accurately, and overestimation and underestimation could be possible in those previous studies. A frequently used method is the documented occupation and answer questionnaire to mark the exposure group and control group [22, 23, 30, 36]. Another method is the geographic division, whose ultraviolet radiation would be the exposure for the groups [17]. Those defects of definition might lead to the overlapping of sunlight exposure. On the other hand, as the living habits are different and the sunlight exposure in the leisure time is also quite different, even some methods could be corrected partly [19]. Finally, with popular sunlight-protecting equipment, such as sunglasses and hat, the exposure of sunlight could not be measured or estimated correctly.

The restriction of our meta-analysis should be noted. The possible underestimation or overestimation of pooled OR could not be ignored due to the various methodological limitations and grouping criteria. And as the language limitation, the eligible studies, only written in English and Chinese, could be included, which might induce a bias. Moreover, the funnel plots in our analysis seemed asymmetrical. Despite this faultiness, the meta-analysis of our study makes an important contribution to the etiology of ARC, as the first study to explore the possible relationship between sunlight exposure and ARC, especially for these three morphological types, cortical, nuclear, and PSC.

## 5. Conclusion

The meta-analysis and systemic review of epidemiological studies published till now revealed that the higher exposure of sunlight would increase the risk of ARC significantly. The advanced analysis of morphological types showed that the high level of sunlight exposure is slightly increased risk on the cortical cataract, but the high sunlight exposure has no risk effect on the nuclear or PSC. The differences in the study population and the methodological quality and criterion of the studies had a potential effect on heterogeneity.

## Figures and Tables

**Table 1:** Joanna Briggs Institute critical appraisal for the cross-sectional study.

Author	1	2	3	4	5	6	7	8	9
McCarty 2000	N	Y	N	Y	Y	N	N	Y	Y
McCarty 1999	Y	Y	N	Y	Y	N	N	Y	Y
West 1998	N	Y	N	Y	Y	N	N	Y	Y
Taylor 1988	N	Y	N	Y	Y	N	N	Y	Y
Delcourt 2000	N	Y	N	Y	Y	N	N	Y	Y
Dherani 2008	N	Y	N	Y	Y	N	N	Y	Y
Hayashi 1998	N	Y	N	Y	Y	N	N	Y	Y
Megbele 2012	N	Y	N	Y	Y	N	N	Y	Y
Moise 2012	N	Y	N	Y	Y	N	N	Y	Y
Paula 2006	N	Y	N	Y	Y	N	N	Y	Y
Tarwadi 2011	N	Y	N	Y	Y	N	N	Y	Y
Delcourt 1993	N	Y	N	Y	Y	N	N	Y	Y
Delavar 2018	N	Y	N	Y	Y	Y	N	Y	Y
Vashist 2020	Y	Y	N	Y	Y	N	N	Y	Y
AREDS 2001	N	Y	N	Y	Y	Y	N	Y	Y
Cruickshanks 1992	N	Y	N	Y	Y	Y	N	Y	Y
Katoh 2001	N	Y	N	Y	Y	Y	N	Y	Y
Klein 2010	N	Y	N	Y	Y	Y	N	Y	Y
Tang 2015	Y	Y	N	Y	Y	Y	N	Y	Y
Miyashita 2019	N	Y	N	Y	Y	Y	N	Y	Y

1: was the study based on a random or pseudorandom sample? 2: were the criteria for inclusion in the sample clearly defined? 3: were confounding factors identified and strategies to deal with them stated? 4: were outcomes assessed using objective criteria? 5: if comparisons are being made, was there sufficient description of the groups? 6: was follow-up carried out over a sufficient time period? 7: were the outcomes of people who withdrew described and included in the analysis? 8: were outcomes measured in a reliable way? 9: was appropriate statistical analysis used? N: no; Y: yes.

**Table 2:** Joanna Briggs Institute critical appraisal for the cohort/case-control study.

Author	1	2	3	4	5	6	7	8	9
Burton 1997	Y	Y	Y	Y	Y	N	N	Y	Y
Collman 1988	N	Y	Y	Y	Y	N	N	Y	Y
Pastor-Valero 2007	N	Y	Y	Y	Y	N	N	Y	Y
Saadat 2006	N	Y	Y	Y	Y	N	N	Y	Y
Sreenivas 1999	Y	Y	Y	Y	Y	N	N	Y	Y
Taylor 1980	N	Y	Y	Y	Y	N	N	Y	Y
Theodoropoulou 2011	N	Y	Y	Y	Y	N	N	Y	Y
Wong 1993	N	Y	Y	Y	Y	N	N	Y	Y
Yu 2016	Y	Y	Y	Y	Y	N	N	Y	Y
Leske1991	N	Y	Y	Y	Y	N	N	Y	Y
Neale 2003	N	Y	Y	Y	Y	N	N	Y	Y

1: is the sample representative of patients in the population as a whole? 2: are the patients at a similar point in the course of their condition/illness? 3: has bias been minimized in relation to selection of cases and of controls? 4: are confounding factors identified and strategies to deal with them stated? 5: are outcomes assessed using objective criteria? 6: was follow-up carried out over a sufficient time period? 7: were the outcomes of people who withdrew described and included in the analysis? 8: were outcomes measured in a reliable way? 9: was appropriate statistical analysis used? N: no; Y: yes.

**Table 3:** The included studies for the meta-analysis of the association between sunlight and age-related cataract (ARC).

Public year	Before 2000	2000 to 2009	2010 to now	Sum
Age-related cataract	7	5	8	20
Cortical cataract	4	5	3	12
Nuclear cataract	5	3	3	11
Posterior capsular cataract	4	2	3	9
Total	11	9	11	31

## Data Availability

The clinic data used to support the findings of this study are included within the article.
